# Poly[(μ-3,5-dinitro-2-oxidobenzoato)(μ-3-hydroxy­pyridine)copper(II)]

**DOI:** 10.1107/S1600536808009392

**Published:** 2008-04-10

**Authors:** Jian-Bin Yan, Wen-Dong Song, Hao Wang, Li-Li Ji

**Affiliations:** a College of Science, Guang Dong Ocean University, Zhanjiang 524088, People’s Republic of China

## Abstract

A new coordination polymer, [Cu(C_7_H_2_N_2_O_7_)(C_5_H_5_NO)]_*n*_, exhibits a double-chain structure, in which 3,5-dinitro-2-oxidobenzoate and 3-hydroxy­pyridine both act as bridging ligands, ­connecting adjacent copper(II) centers to form an infinite double-stranded chain. The asymmetric unit contains one Cu^II^ ion, one 3,5-dinitro-2-oxidobenzoate ligand and a 3-hydroxy­pyridine ligand. Coordination by one N atom and three O atoms from two different 3,5-dinitro-2-oxidobenzoate ligands and a 3-hydroxy­pyridine ligand creates a square-planar Cu^II^ center, which is augmented by a less tightly bonded fifth phenol O atom to form a square-pyramidal five-coordinate complex with an essentially planar base. The double-stranded chains are stabilized by intra­chain π–π inter­actions [the centroid-to-centroid distance between adjacent aromatic rings is 3.719 (7) Å], and further linked through O—H⋯O hydrogen bonds, forming a three-dimensional supra­molecular network.

## Related literature

For related literature, see: Bradshaw *et al.* (2005 ▶); Eddaoudi *et al.* (2001 ▶); Fujita *et al.* (1994 ▶); Gable *et al.* (1990 ▶); Gao *et al.* (2005 ▶); He *et al.* (2006 ▶); Li *et al.* (1999 ▶); Losier & Zaworotko (1996 ▶); Moulton & Zaworotko (2001 ▶); Song & Xi (2006 ▶) Song *et al.*, 2006 ▶; Song, Guo & Guo, 2007 ▶; Song, Guo & He, 2007 ▶; Song, Guo & Zhang, 2007 ▶; Song, Yan *et al.*, 2007 ▶); Stang & Olenyuk (1997 ▶); Withersby *et al.* (1999 ▶); Yaghi & Li (1995 ▶).
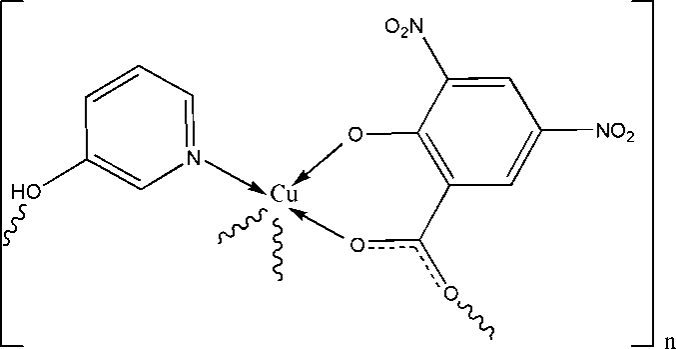


## Experimental

### 

#### Crystal data

[Cu(C_7_H_2_N_2_O_7_)(C_5_H_5_NO)]
*M* *_r_* = 384.75
Monoclinic, 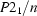

*a* = 8.1055 (3) Å
*b* = 6.2208 (2) Å
*c* = 26.9837 (9) Å
β = 94.030 (3)°
*V* = 1357.23 (8) Å^3^
*Z* = 4
Mo *K*α radiation
μ = 1.66 mm^−1^
*T* = 296 (2) K
0.25 × 0.16 × 0.09 mm

#### Data collection

Bruker APEXII area-detector diffractometer
Absorption correction: multi-scan (*SADABS*; Sheldrick, 1996 ▶) *T*_min_ = 0.681, *T*_max_ = 0.865
12917 measured reflections
3094 independent reflections
2275 reflections with *I* > 2σ(*I*)
*R*_int_ = 0.061

#### Refinement

*R*[*F* ^2^ > 2σ(*F* ^2^)] = 0.040
*wR*(*F* ^2^) = 0.092
*S* = 1.03
3094 reflections
218 parameters
H-atom parameters constrained
Δρ_max_ = 0.38 e Å^−3^
Δρ_min_ = −0.45 e Å^−3^

### 

Data collection: *APEX2* (Bruker, 2004 ▶); cell refinement: *SAINT* (Bruker, 2004 ▶); data reduction: *SAINT*; program(s) used to solve structure: *SHELXS97* (Sheldrick, 2008 ▶); program(s) used to refine structure: *SHELXL97* (Sheldrick, 2008 ▶); molecular graphics: *DIAMOND* (Brandenburg, 2001 ▶); software used to prepare material for publication: *SHELXTL* (Sheldrick, 2008 ▶).

## Supplementary Material

Crystal structure: contains datablocks I, global. DOI: 10.1107/S1600536808009392/zl2099sup1.cif

Structure factors: contains datablocks I. DOI: 10.1107/S1600536808009392/zl2099Isup2.hkl

Additional supplementary materials:  crystallographic information; 3D view; checkCIF report

## Figures and Tables

**Table 1:** Hydrogen-bond geometry (Å, °)

*D*—H⋯*A*	*D*—H	H⋯*A*	*D*⋯*A*	*D*—H⋯*A*
O8—H8*A*⋯O4^i^	0.82	1.94	2.738 (3)	165

Symmetry code: (i) 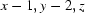.

**Fig. 1.**
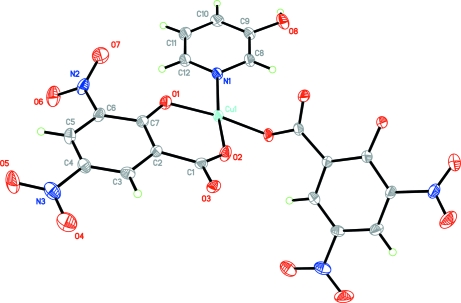
The structure of (I), showing the atom-numbering scheme and displacement ellipsoids drawn at the 30% probability level. Unlabeled atoms are related to the labelled atoms by the symmetry operator (x, 0.5 - y, -1/2 + z).

**Fig. 2.**
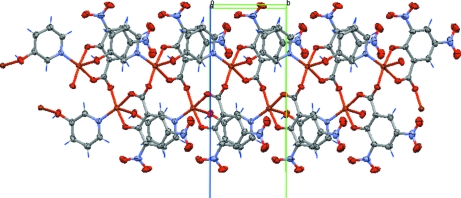
One double stranded chain of (I).

**Fig. 3.**
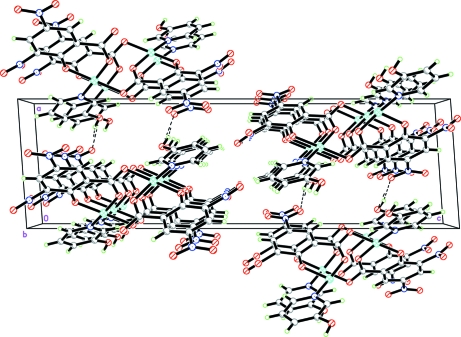
A packing view of (I) along the b axis. Hydrogen bonds are depicted as broken lines.

## supplementary crystallographic information

### Comment

Inorganic-organic coordination polymers have been a focus of contemporary research interest not only because of the intriguing variety of architectures and topologies but also because of their potential application in gas storage, catalysis, as molecular magnets, in molecular recognition and photoluminescene (Stang & Olenyuk,1997; Moulton & Zaworotko, 2001; Eddaoudi *et al.*, 2001; Bradshaw *et al.*, 2005). Over the past few decades considerable efforts have documented many networks with various structural motifs including honeycomb, brick-wall, rectangular grid, bilayer, ladder, herringbone, diamondoid and octahedral geometries (Gable *et al.*, 1990; Fujita *et al.*, 1994; Yaghi & Li, 1995; Losier & Zaworotko, 1996; Withersby *et al.*, 1999; Li *et al.*, 1999). Building blocks based on aromatic acids, such as benzoic acid and/or its substituted derivatives, have been used in the construction of coordination polymers. However, there are few examples of metal derivatives of 3,5-dinitrosalicylic acid, and examples of crystal structure reports are limited to silver and tin complexes only. 3,5-Dinitrosalicylic acid is, owing to its versatile coordination modes, an important multidentate ligand for metal complexes (He *et al.*, 2006). Recently, some new structures with the 3,5-dinitrosalicylicate have been synthesized by our group (Song & Xi (2006), 2006; Song, Guo & Guo, 2007; Song, Guo & He, 2007; Song,Guo & Zhang, 2007;  Song, Yan *et al.*, 2007).

With the intention of studying the influences of aromatic bridging ligands on the frameworks of possible structures, we chose another non-chelating ligand, 3-hydroxyprridine, as a second ligand (Gao *et al.*, 2005), and a new coordination polymer, [Cu(C_7_H_3_N_2_O_7_)(C_5_H_5_NO)]_n_, (I), was successfully synthesized.

The coordination environment of the copper center in complex (I) is depicted in Fig. 1. The asymmetric unit contains one Cu^II^ ion, one 3,5-dinitro-2-oxidobenzoate ligand and a 3-hydroxypyridine ligand. Coordination by one N atom and three O atoms from two different 3,5-dinitro-2-oxidobenzoate ligands and 3-hydroxypyridine ligand create a square planar Cu^II^ center, which is augmented by a less tightly bonded fifth phenol oxygen atom to form a square pyramidal five-coordinated complex with a basically flat base. The compound exhibits a double chain structure, in which the 3,5-dinitro-2-oxidobenzoate and 3-hydroxypyridine both act as bridging ligands interconnecting adjacent copper(II) centers to form an infinite double stranded chain along the *b* axis of the unit cell (Fig 2.). These double stranded chains are stabilized by intra-chain π-π interactions, and further linked through O—H···O hydrogen bonding interaction involving the hydroxyl group of the 3-hydroxypyridine ligand as the H-donor and a nitryl group of the 3,5-dinitro-2-oxidobenzoate ligand as the acceptor, thus forming a three-dimensional supramolecular network (Fig. 3). The centroid-to-centroid distance of adjacent aromatic rings is 3.719 (7) Å.

### Experimental

A mixture of CuCl_2_ (0.134 g; 1 mmol), 3,5-dinitrosalicylic acid (0.228 g; 1 mmol), 3-hydroxypyridine (0.095 g; 1 mmol) and H_2_O (10 ml) was stirred vigorously for 20 min and then sealed in a Teflon-lined stainless-steel autoclave (20 ml, capacity). The autoclave was heated to and maintained at 433 K for 5 days, and then cooled to room temperature at 5 K h^-1^. The blue plate single crystals were obtained in *ca* 78% yield based on CuCl_2_.

### Refinement

Aromatic and hydroxyl H atoms were placed in calculated positions and were treated as riding on the parent C atoms with C—H = 0.93 Å, O—H = 0.82 Å (hydroxyl group) and with *U*_iso_(H) = 1.2 or 1.5 *U*_eq_(C, O). Hydroxyl H atoms were allowed to rotate around the C—O direction to best fit the experimental electron denisty.

### Crystal data

**Table:**

[Cu(C_7_H_2_N_2_O_7_)(C_5_H_5_NO)]	*F*_000_ = 772
*M**_r_* = 384.75	*D*_x_ = 1.883 Mg m^−^^3^
Monoclinic, *P*2_1_/*n*	Mo *K*α radiation λ = 0.71073 Å
Hall symbol: -P 2yn	Cell parameters from 2895 reflections
*a* = 8.1055 (3) Å	θ = 2.4–27.9º
*b* = 6.2208 (2) Å	µ = 1.66 mm^−^^1^
*c* = 26.9837 (9) Å	*T* = 296 (2) K
β = 94.030 (3)º	Plate, blue
*V* = 1357.23 (8) Å^3^	0.25 × 0.16 × 0.09 mm
*Z* = 4	

### Data collection

**Table:**

Bruker APEXII area-detector diffractometer	3094 independent reflections
Radiation source: fine-focus sealed tube	2275 reflections with *I* > 2σ(*I*)
Monochromator: graphite	*R*_int_ = 0.061
*T* = 296(2) K	θ_max_ = 27.5º
φ and ω scans	θ_min_ = 2.6º
Absorption correction: multi-scan(SADABS; Sheldrick, 1996)	*h* = −10→10
*T*_min_ = 0.681, *T*_max_ = 0.865	*k* = −8→8
12917 measured reflections	*l* = −31→34

### Refinement

**Table:**

Refinement on *F*^2^	Secondary atom site location: difference Fourier map
Least-squares matrix: full	Hydrogen site location: inferred from neighbouring sites
*R*[*F*^2^ > 2σ(*F*^2^)] = 0.040	H-atom parameters constrained
*wR*(*F*^2^) = 0.092	*w* = 1/[σ^2^(*F*_o_^2^) + (0.0416*P*)^2^] where *P* = (*F*_o_^2^ + 2*F*_c_^2^)/3
*S* = 1.03	(Δ/σ)_max_ < 0.001
3094 reflections	Δρ_max_ = 0.38 e Å^−^^3^
218 parameters	Δρ_min_ = −0.45 e Å^−^^3^
Primary atom site location: structure-invariant direct methods	Extinction correction: none

### Special details

**Table:**

Geometry. All e.s.d.'s (except the e.s.d. in the dihedral angle between two l.s. planes) are estimated using the full covariance matrix. The cell e.s.d.'s are taken into account individually in the estimation of e.s.d.'s in distances, angles and torsion angles; correlations between e.s.d.'s in cell parameters are only used when they are defined by crystal symmetry. An approximate (isotropic) treatment of cell e.s.d.'s is used for estimating e.s.d.'s involving l.s. planes.
Refinement. Refinement of *F*^2^ against ALL reflections. The weighted *R*-factor *wR* and goodness of fit *S* are based on *F*^2^, conventional *R*-factors *R* are based on *F*, with *F* set to zero for negative *F*^2^. The threshold expression of *F*^2^ > σ(*F*^2^) is used only for calculating *R*-factors(gt) *etc*. and is not relevant to the choice of reflections for refinement. *R*-factors based on *F*^2^ are statistically about twice as large as those based on *F*, and *R*- factors based on ALL data will be even larger.

### Fractional atomic coordinates and isotropic or equivalent isotropic displacement parameters (Å^2^)

**Table:**

	*x*	*y*	*z*	*U*_iso_*/*U*_eq_	
Cu1	0.12663 (5)	0.27589 (5)	0.187438 (14)	0.02720 (13)	
O1	0.2121 (3)	0.3893 (3)	0.12851 (7)	0.0336 (5)	
C3	0.4627 (4)	0.8652 (4)	0.15767 (12)	0.0300 (7)	
H3	0.5168	0.9353	0.1846	0.036*	
C7	0.2967 (4)	0.5637 (4)	0.12445 (11)	0.0266 (6)	
C2	0.3785 (4)	0.6762 (4)	0.16517 (11)	0.0257 (6)	
C6	0.3114 (4)	0.6587 (4)	0.07688 (11)	0.0303 (7)	
C5	0.3921 (4)	0.8497 (5)	0.06963 (12)	0.0339 (7)	
H5	0.3957	0.9089	0.0381	0.041*	
C4	0.4673 (4)	0.9503 (4)	0.11085 (12)	0.0319 (7)	
N2	0.2313 (3)	0.5572 (4)	0.03232 (10)	0.0383 (7)	
N3	0.5560 (3)	1.1514 (4)	0.10454 (13)	0.0432 (7)	
O5	0.5735 (3)	1.2190 (4)	0.06299 (10)	0.0593 (8)	
O4	0.6093 (3)	1.2453 (3)	0.14266 (11)	0.0569 (7)	
O6	0.1611 (3)	0.6731 (4)	0.00078 (10)	0.0580 (7)	
O7	0.2381 (4)	0.3631 (4)	0.02875 (9)	0.0591 (7)	
N1	0.0150 (3)	0.0378 (3)	0.14852 (9)	0.0280 (6)	
C10	−0.1449 (4)	−0.2943 (4)	0.09616 (13)	0.0354 (8)	
H10	−0.1983	−0.4056	0.0785	0.043*	
C8	−0.0260 (4)	−0.1437 (4)	0.17174 (12)	0.0308 (7)	
H8	−0.0007	−0.1566	0.2058	0.037*	
C9	−0.1048 (4)	−0.3115 (4)	0.14630 (12)	0.0294 (7)	
C12	−0.0227 (4)	0.0547 (4)	0.09966 (12)	0.0335 (7)	
H12	0.0064	0.1790	0.0832	0.040*	
C11	−0.1036 (4)	−0.1078 (5)	0.07304 (13)	0.0377 (8)	
H11	−0.1303	−0.0907	0.0392	0.045*	
O8	−0.1323 (3)	−0.4927 (3)	0.17369 (9)	0.0429 (6)	
H8A	−0.2046	−0.5657	0.1591	0.064*	
C1	0.3686 (4)	0.6059 (4)	0.21742 (11)	0.0272 (7)	
O2	0.2620 (3)	0.4681 (3)	0.22901 (8)	0.0378 (5)	
O3	0.4641 (2)	0.6940 (3)	0.25017 (8)	0.0290 (5)	

### Atomic displacement parameters (Å^2^)

**Table:**

	*U*^11^	*U*^22^	*U*^33^	*U*^12^	*U*^13^	*U*^23^
Cu1	0.0369 (2)	0.02288 (17)	0.0209 (2)	−0.00557 (14)	−0.00425 (16)	0.00164 (14)
O1	0.0472 (14)	0.0299 (10)	0.0231 (12)	−0.0138 (9)	−0.0003 (10)	−0.0021 (8)
C3	0.0300 (17)	0.0254 (13)	0.034 (2)	−0.0014 (11)	−0.0011 (14)	−0.0047 (12)
C7	0.0291 (17)	0.0262 (13)	0.0241 (17)	−0.0016 (11)	−0.0008 (13)	−0.0019 (11)
C2	0.0298 (17)	0.0255 (13)	0.0215 (17)	−0.0004 (11)	−0.0002 (13)	−0.0009 (11)
C6	0.0342 (18)	0.0365 (15)	0.0195 (18)	−0.0052 (12)	−0.0021 (14)	−0.0005 (12)
C5	0.0335 (19)	0.0391 (15)	0.0294 (19)	−0.0064 (13)	0.0038 (15)	0.0076 (14)
C4	0.0291 (17)	0.0282 (13)	0.038 (2)	−0.0066 (12)	0.0038 (15)	0.0039 (13)
N2	0.0425 (17)	0.0507 (16)	0.0216 (16)	−0.0148 (13)	0.0006 (13)	−0.0006 (12)
N3	0.0349 (17)	0.0343 (13)	0.060 (2)	−0.0077 (12)	0.0038 (16)	0.0058 (14)
O5	0.0653 (19)	0.0535 (14)	0.060 (2)	−0.0221 (12)	0.0112 (15)	0.0213 (13)
O4	0.0575 (17)	0.0447 (13)	0.068 (2)	−0.0267 (11)	0.0005 (15)	−0.0063 (12)
O6	0.0634 (19)	0.0757 (17)	0.0321 (16)	−0.0060 (14)	−0.0154 (13)	0.0153 (13)
O7	0.090 (2)	0.0474 (14)	0.0379 (17)	−0.0132 (13)	−0.0070 (15)	−0.0116 (12)
N1	0.0331 (15)	0.0236 (11)	0.0265 (15)	−0.0028 (9)	−0.0037 (11)	0.0018 (10)
C10	0.0395 (19)	0.0282 (14)	0.037 (2)	−0.0087 (12)	−0.0082 (16)	−0.0089 (13)
C8	0.0362 (18)	0.0258 (13)	0.0291 (19)	−0.0049 (12)	−0.0072 (15)	0.0043 (12)
C9	0.0279 (17)	0.0246 (13)	0.035 (2)	−0.0017 (11)	−0.0038 (14)	0.0030 (12)
C12	0.0430 (19)	0.0294 (14)	0.0269 (19)	−0.0071 (13)	−0.0052 (15)	−0.0006 (12)
C11	0.045 (2)	0.0382 (16)	0.0282 (19)	−0.0047 (14)	−0.0052 (16)	−0.0036 (14)
O8	0.0481 (16)	0.0281 (10)	0.0501 (16)	−0.0143 (9)	−0.0124 (12)	0.0091 (10)
C1	0.0321 (17)	0.0246 (13)	0.0242 (18)	0.0024 (11)	−0.0024 (14)	−0.0046 (11)
O2	0.0511 (15)	0.0399 (11)	0.0214 (12)	−0.0187 (10)	−0.0040 (10)	0.0005 (9)
O3	0.0315 (12)	0.0313 (10)	0.0231 (12)	−0.0031 (8)	−0.0055 (9)	−0.0073 (8)

### Geometric parameters (Å, °)

**Table:**

Cu1—O1	1.9132 (19)	N3—O5	1.215 (4)
Cu1—O2	1.929 (2)	N3—O4	1.234 (4)
Cu1—O3^i^	1.952 (2)	N1—C12	1.336 (4)
Cu1—N1	1.996 (2)	N1—C8	1.344 (3)
O1—C7	1.292 (3)	C10—C11	1.370 (4)
C3—C4	1.373 (4)	C10—C9	1.373 (5)
C3—C2	1.382 (4)	C10—H10	0.9300
C3—H3	0.9300	C8—C9	1.381 (4)
C7—C6	1.426 (4)	C8—H8	0.9300
C7—C2	1.426 (4)	C9—O8	1.375 (3)
C2—C1	1.484 (4)	C12—C11	1.379 (4)
C6—C5	1.377 (4)	C12—H12	0.9300
C6—N2	1.468 (4)	C11—H11	0.9300
C5—C4	1.380 (4)	O8—H8A	0.8200
C5—H5	0.9300	C1—O3	1.259 (3)
C4—N3	1.459 (3)	C1—O2	1.271 (3)
N2—O7	1.213 (3)	O3—Cu1^ii^	1.952 (2)
N2—O6	1.224 (3)		
			
O1—Cu1—O2	91.74 (8)	O5—N3—O4	123.3 (3)
O1—Cu1—O3^i^	173.49 (8)	O5—N3—C4	119.7 (3)
O2—Cu1—O3^i^	83.90 (8)	O4—N3—C4	117.1 (3)
O1—Cu1—N1	90.78 (9)	C12—N1—C8	118.6 (2)
O2—Cu1—N1	169.99 (10)	C12—N1—Cu1	121.62 (18)
O3^i^—Cu1—N1	94.34 (9)	C8—N1—Cu1	119.7 (2)
C7—O1—Cu1	127.25 (19)	C11—C10—C9	117.8 (3)
C4—C3—C2	120.6 (3)	C11—C10—H10	121.1
C4—C3—H3	119.7	C9—C10—H10	121.1
C2—C3—H3	119.7	N1—C8—C9	121.6 (3)
O1—C7—C6	120.2 (3)	N1—C8—H8	119.2
O1—C7—C2	124.6 (3)	C9—C8—H8	119.2
C6—C7—C2	115.2 (2)	C10—C9—O8	123.9 (3)
C3—C2—C7	120.8 (3)	C10—C9—C8	120.0 (3)
C3—C2—C1	116.8 (3)	O8—C9—C8	116.1 (3)
C7—C2—C1	122.3 (2)	N1—C12—C11	121.6 (3)
C5—C6—C7	123.8 (3)	N1—C12—H12	119.2
C5—C6—N2	116.2 (3)	C11—C12—H12	119.2
C7—C6—N2	119.9 (2)	C10—C11—C12	120.4 (3)
C6—C5—C4	117.7 (3)	C10—C11—H11	119.8
C6—C5—H5	121.2	C12—C11—H11	119.8
C4—C5—H5	121.2	C9—O8—H8A	109.5
C3—C4—C5	121.8 (3)	O3—C1—O2	121.0 (3)
C3—C4—N3	118.9 (3)	O3—C1—C2	117.9 (3)
C5—C4—N3	119.2 (3)	O2—C1—C2	121.0 (3)
O7—N2—O6	123.5 (3)	C1—O2—Cu1	130.1 (2)
O7—N2—C6	118.2 (3)	C1—O3—Cu1^ii^	117.95 (19)
O6—N2—C6	118.2 (3)		

Symmetry codes: (i) −*x*+1/2, *y*−1/2, −*z*+1/2; (ii) −*x*+1/2, *y*+1/2, −*z*+1/2.

### Hydrogen-bond geometry (Å, °)

**Table:**

*D*—H···*A*	*D*—H	H···*A*	*D*···*A*	*D*—H···*A*
O8—H8A···O4^iii^	0.82	1.94	2.738 (3)	165

Symmetry codes: (iii) *x*−1, *y*−2, *z*.
